# Long-term clinical outcomes after coronary artery bypass grafting with pedicled saphenous vein grafts

**DOI:** 10.1186/s13019-018-0800-z

**Published:** 2018-11-26

**Authors:** Mikael Janiec, Örjan Friberg, Stefan Thelin

**Affiliations:** 1 Department of Cardiothoracic Surgery and Anaesthesia, Uppsala University Hospital, SE-751 85 Uppsala, Sweden; 2 Department of Surgical Sciences, Section of Thoracic Surgery, Uppsala University, Uppsala, Sweden; 3 Department of Cardiothoracic and Vascular Surgery, Faculty of Medicine and Health, Örebro University, SE-701 82 Örebro, Sweden

**Keywords:** CABG, Coronary artery bypass grafting, No-touch, Pedicled vein grafts

## Abstract

**Background:**

Coronary artery bypass grafting (CABG) using saphenous vein grafts (SVG) is vitiated by poor long-term patency of the vein grafts. Pedicled SVG harvested with the “no-touch” (NT) technique have demonstrated improved patency and could confer better outcomes. We aim to compare long-term results after CABG where NT or conventional technique was used for vein graft harvesting in a hypothesis-generating registry-based study.

**Methods:**

Two propensity score matched cohorts (1349 patients) undergoing CABG with veins harvested with NT (NTT) or conventional (CT) technique in Sweden over the period 2005–2015 were used to compare long-term outcomes. Mortality, postoperative incidence of coronary angiography and need for reintervention was recorded and multivariable hazard ratios adjusted for risk factors were calculated.

**Results:**

The mean follow-up time (SD) was 6.8 (3.3) years for NTT and 6.6 (3.2) years for CT. The adjusted hazard ratios for death, first angiography and need for reintervention for NTT patients were (95% CI) 0.97 (0.80–1.19), 0.76 (0.63–0.93), 0.91 (0.78–1.05), and 0.91 (0.71–1.17), respectively. Failed grafts were found in 43.2% of NTT patients and 53.6% of CT patients at angiography.

**Conclusions:**

In this study NT grafting was associated with a lower risk for repeat angiography, however no difference could be observed for mortality and need for reintervention. The earlier reported improvements in patency of NT veins could possibly be reflected in an improved clinical outcome during the first 10 years after surgery.

**Electronic supplementary material:**

The online version of this article (10.1186/s13019-018-0800-z) contains supplementary material, which is available to authorized users.

## Background

Coronary artery bypass grafting (CABG) is the recommended treatment for coronary artery disease (CAD) involving complex lesions in multiple vessels, with a survival benefit compared to percutaneous coronary intervention (PCI) [1–4]. Saphenous vein grafts (SVG) are most often used but are subject to graft disease [5] and their reduced long-term patency compared to left internal mammary artery (IMA) grafts is well established [6, 7]. The success of CABG depends arguably on the long-term patency rate of the conduits and several risk factors for graft failure have been identified [8–10]. Although most risk factors are related to the target vessel and cannot be influenced, damage to graft vessels during surgical preparation and dilation has also been shown to influence patency [11]. More than 20 years ago Souza et al. initiated studies of vein grafts harvested with a pedicle of surrounding tissue and without distending the grafts [12]. This “no-touch” (NT) technique is believed to preserve the vein vasa vasora [13] and the grafts exhibit a slower progression of intimal hyperplasia [14] and atherosclerosis [15]. A single-center randomized clinical trial with angiographic follow-up of these grafts from has demonstrated significantly higher patency for NT harvested veins compared with veins harvested with the conventional technique [16] as well as with radial artery grafts [17]. The patency of NT grafts was comparable to that of the IMA still 16 years after surgery [18]. The influence on clinical outcome, that the presumably improved patency of grafts harvested with the NT technique could entail, has however not been studied.

The Swedish personal identity number allows nationwide registries with the possibility of long-term follow-up. The Swedish Web System for Enhancement and Development of Evidence-Based Care in Heart Disease Evaluated According to Recommended Therapies (SWEDEHEART) registry [19, 20] provides data on all patients undergoing cardiac surgery in Sweden as well as records of angiographies and coronary interventions. Mortality yields limited information when used as sole outcome in studies of graft patency, as it is most probably only to a lesser degree influenced by the effects of failing grafts after CABG surgery [8]. In the present study we use incidence of clinically-driven angiography and need for repeat intervention as endpoints, in addition to mortality, for evaluation of long-term results after CABG. These outcomes are likely to have a high association with symptomatic graft failure and related events and have been used previously to evaluate results after CABG [21]. Cardiac surgeons in Örebro have implemented the NT practice in a majority of cases since more than 10 years whereas vein grafts are harvested with a conventional technique in other centres in Sweden. Data on vein harvesting technique for patients operated in Örebro is available in a local registry. Here, we evaluate outcomes in the more than 1300 patients operated with CABG using NT vein grafts during the last 10 years in an observational propensity matched cohort study. We aim to quantify the mortality, incidence of first angiography and need for repeat intervention and evaluate possible differences in between patients operated in Örebro with the NT technique and Swedish patients operated with the conventional technique.

## Methods

### Data sources and study population

**Fig. 1:**
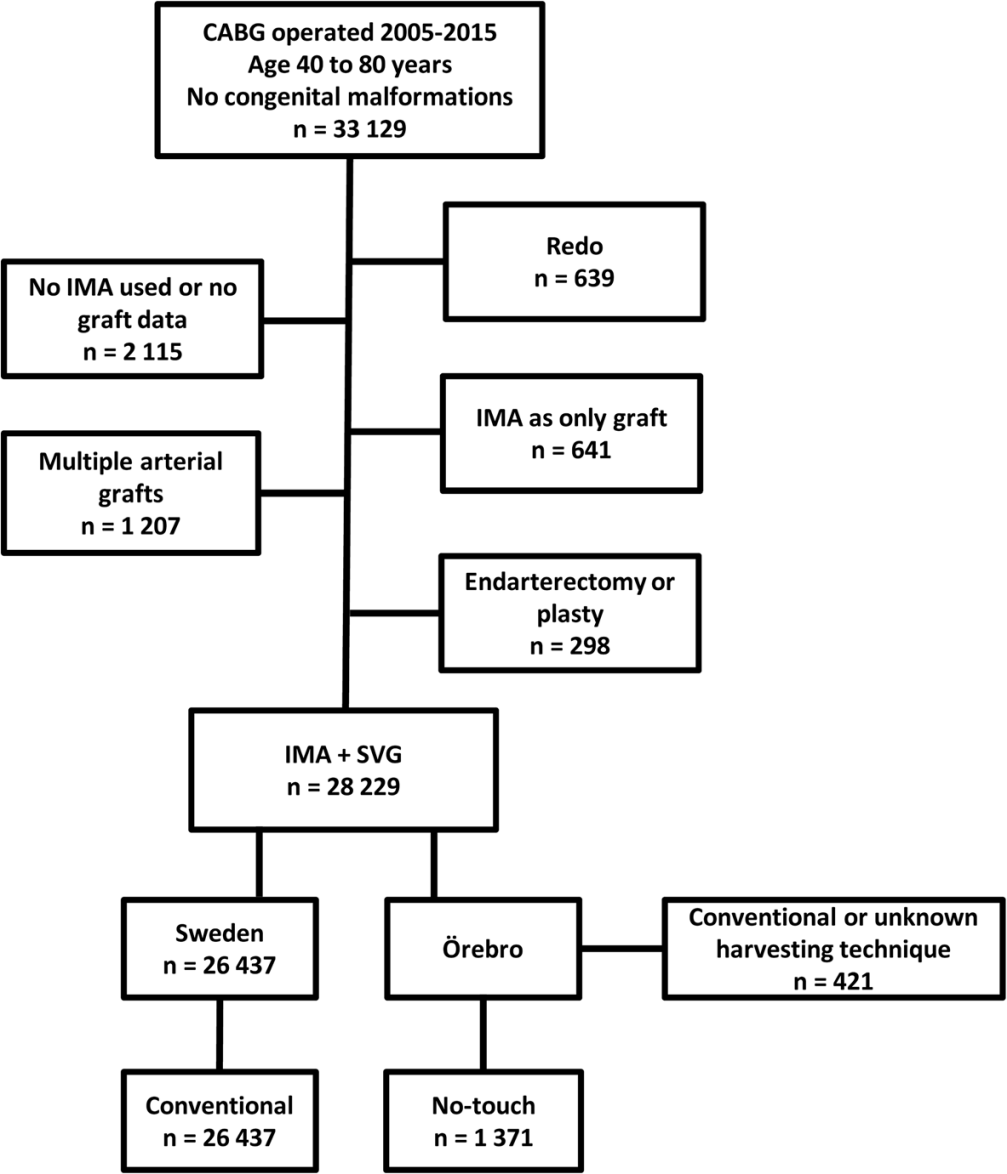
Flow chart of patients. All CABG operated patients in Sweden between 2005 and 2015, aged 40 to 80 years old, without congenital malformations, were included in the study data base. We excluded redo operations, cases where no IMA was used or where graft information was missing, cases where single IMA was the only graft used, cases where arterial grafts other than the single IMA were used and cases where an endarterectomy or plasty was performed. CABG indicates coronary artery bypass grafting; IMA internal mammary artery; SVG saphenous vein graft

The study was approved by the regional Human Research Ethics Committee, Uppsala, Sweden. Patients between 40 and 80 years of age with no congenital malformations and with a permanent residence in Sweden, who underwent isolated CABG in Sweden between 2005 and 2015, were identified within the SWEDEHEART registry. We excluded patients previously having undergone cardiac surgery, where no IMA was used or the types of grafts were unknown, cases operated with IMA as the only graft, cases where multiple arterial grafts were used or in which coronary endarterectomy was performed. The remaining cohort consisted of patients with a single IMA and one or more SVG. The type of technique used for vein harvesting for patients operated in Örebro was obtained from a local registry (Fig. 1). We obtained baseline characteristics at time of surgery and data on all postoperative angiographies for included individuals.

### Outcomes

Date of death was obtained from the national population registry. The dates for all operations and postoperative angiographies were available in SWEDEHEART. For each angiography the indication for the procedure, the presence of significantly stenosed or occluded grafts and the subsequent therapeutic decision, including recommendations for revascularization, is registered by the angiographer. The elapsed time from the operation to death, first clinically-driven angiography or first need for reintervention was used as endpoints. The only patients that are believed to have been lost to follow-up are those having gone through angiography or reintervention outside of Sweden.

### Statistical methods

Patient characteristics were described by using frequencies and percentages for categorical variables, and means and standard deviations for continuous variables. The outcome measures were evaluated in the population as the time from operation to death from any cause, to first angiography after surgery and to the first need for reintervention after surgery. Patients were followed from the date of surgery until the date of death from any cause or the end of follow-up (May 05, 2016). Data management and statistical analyses were performed with the use of R version 3.1.3 (R Foundation for Statistical Computing, Vienna, Austria). The cohort was divided into two groups according to the graft harvesting technique:

1. **NTT**, patients operated in Örebro where SVG were harvested with NT technique.
2. **CT**, patients operated at other departments in Sweden, operated with the conventional technique for vein harvesting.

Propensity score (PS) matching was used to reduce the effect of treatment-selection bias using a binary dependent variable representing the vein harvesting technique. Independent variables included year of surgery, age (40–50, 50–60, 50–65, 65–70, 70–75 or 75–80 years), sex, number of distal anastomoses (2, 3, 4, or 5+), body mass index (BMI) (< 25, 25–30 or > 30), presence of diabetes, renal function (creatinine clearance > 85, 50–85, < 50 or dialysis), chronic obstructive pulmonary disease (COPD), peripheral vascular disease, and neurologic disability, left ventricle ejection fraction (normal, 30–50% or < 30%), prior myocardial infarction, previous percutaneous coronary intervention (PCI), emergency surgery (beginning before next working day after decision) and surgery without the use of cardiopulmonary bypass. Patients with missing data were assumed to be missing at random and excluded from the matching. The matching procedure was performed by the “MatchIt” R package and using “nearest neighbour matching” and the propensity score logit as distance measure to select the best control matches for each individual in the NTT group. In order to assess imbalances in baseline covariates after matching standardized differences were used, where differences of < 0.10 are likely to indicate a negligible imbalance between the two groups. The Kaplan-Meier method was used to illustrate cumulative survival and cumulative incidence of first clinically-driven postoperative angiography and first need for reintervention. Cox regression was used to estimate the associations of the vein harvest technique with all-cause mortality, incidence of first angiography and need for repeat coronary intervention. The hazard ratios and 95% confidence intervals (CIs) for the three groups were calculated unadjusted and adjusted for baseline covariates with the ‘survival’ R package. Smoothed scaled Schoenfeld residual plots were used to assess the proportional hazards assumption graphically for each variable, and no important violation was found. Indications for the first angiography as well as the presence of stenosed grafts were described by using frequencies and percentages. *P*-values of < 0.05 and CI of 95% were used to establish a statistically significant difference between groups.

## Results

### Study population and baseline characteristics

In total, 33,129 Swedish residents between 40 and 80 years of age without congenital heart malformations underwent isolated CABG in Sweden between January 2005 and December 2015. After exclusion the study population was composed of 1371 patients with single IMA and no-touch grafts and 26,437 patients with single IMA and conventional vein grafts (Fig. 1). After PS matching a cohort of 1349 patients in each group was created with standardized differences of covariates < 0.10. The mean follow-up was 6.8 (3.3) years in the NTT group and 6.6 (3.2) years in the CT group. Baseline characteristics are shown in Table 1.

**Table 1:** Baseline statistics and early postoperative complications of the two groups of CABG operated patients before and after matching

			Propensity score-matched cohort		
	Conventional	No-touch	Conventional	No-touch	Standardized differences
Study population	26,437	1371	1349	1349	
Year of surgery:					
2005–2006	6240 (23.6%)	381 (27.8%)	345 (25.6%)	381 (28.2%)	0.06
2007–2008	5856 (22.2%)	283 (20.6%)	303 (22.5%)	283 (21.0%)	0.04
2009–2010	4780 (18.1%)	231 (16.8%)	232 (17.2%)	226 (16.8%)	0.01
2011–2012	4149 (15.7%)	181 (13.2%)	164 (12.2%)	174 (12.9%)	0.02
2013–2015	5412 (20.5%)	295 (21.5%)	305 (22.6%)	285 (21.1%)	0.04
Age (years):					
40–50	1042 (3.9%)	47 (3.4%)	35 (2.6%)	46 (3.4%)	0.05
50–60	4715 (17.8%)	251 (18.3%)	234 (17.3%)	244 (18.1%)	0.02
60–65	4734 (17.9%)	256 (18.7%)	255 (18.9%)	254 (18.8%)	0.00
65–70	5659 (21.4%)	313 (22.8%)	310 (23.0%)	307 (22.8%)	0.01
70–75	5740 (21.7%)	316 (23.0%)	305 (22.6%)	311 (23.1%)	0.01
75–80	4547 (17.2%)	188 (13.7%)	210 (15.6%)	187 (13.9%)	0.05
Female	4853 (18.4%)	207 (15.1%)	197 (14.6%)	201 (14.9%)	0.01
BMI:					
< 25	6903 (28.4%)	373 (27.2%)	387 (28.7%)	365 (27.1%)	0.04
25–30	11,631 (47.8%)	674 (49.2%)	664 (49.2%)	668 (49.5%)	0.01
> 30	5785 (23.8%)	323 (23.6%)	298 (22.1%)	316 (23.4%)	0.03
BMI not recorded	2118 (8.0%)	1 (0.1%)			
Diabetes	6757 (26.1%)	329 (24.0%)	315 (23.4%)	321 (23.8%)	0.01
Presence of diabetes not recorded	584 (2.2%)	1 (0.1%)			
Renal impairment:					
Normal, (CC > 85 ml/min)	12,149 (50.1%)	742 (54.2%)	691 (51.2%)	729 (54.0%)	0.06
Moderately impaired (50–85 ml/min)	10,248 (42.3%)	552 (40.3%)	585 (43.4%)	546 (40.5%)	0.06
Severely impaired (< 50 ml/min) off dialysis	1596 (6.6%)	69 (5.0%)	71 (5.3%)	68 (5.0%)	0.01
On dialysis	253 (1.0%)	6 (0.4%)	2 (0.2%)	6 (0.4%)	0.05
Renal impairment not recorded	2191 (8.3%)	2 (0.2%)			
COPD	1872 (7.3%)	58 (4.3%)	56 (4.2%)	58 (4.3%)	0.01
COPD not recorded	961 (3.6%)	12 (0.9%)			
Extracardiac arteriopathy	2166 (8.3%)	88 (6.4%)	82 (6.1%)	88 (6.5%)	0.02
Extracardiac arteriopathy not recorded	305 (1.2%)	5 (0.4%)			
Neurological disability	554 (2.2%)	45 (3.3%)	44 (3.3%)	45 (3.3%)	0.00
Neurological disability not recorded	971 (3.7%)	1 (0.1%)			
Ejection fraction:					
Normal	18,161 (69.5%)	1055 (77.1%)	1065 (78.9%)	1046 (77.5%)	0.03
30–50%	6682 (25.6%)	259 (18.9%)	242 (17.9%)	251 (18.6%)	0.02
< 30%	1289 (4.9%)	54 (3.9%)	42 (3.1%)	52 (3.9%)	0.04
Ejection fraction not recorded	305 (1.2%)	3 (0.2%)			
MI last 90 days	10,853 (41.5%)	500 (36.5%)	492 (36.5%)	487 (36.1%)	0.01
Prior MI not recorded	304 (1.1%)	0 (0.0%)			
Previous PCI	3537 (16.1%)	269 (19.7%)	254 (18.8%)	263 (19.5%)	0.02
Previous PCI not recorded	4492 (17.0%)	5 (0.4%)			
Emergency surgery	1124 (4.3%)	53 (3.9%)	47 (3.5%)	49 (3.6%)	0.01
Urgency not recorded	62 (0.2%)	0 (0.0%)			
Number of distal anastomoses:					
2	5565 (21.1%)	187 (13.6%)	202 (15.0%)	183 (13.6%)	0.04
3	11,605 (43.9%)	458 (33.4%)	467 (34.6%)	451 (33.4%)	0.03
4	7019 (26.5%)	532 (38.8%)	469 (34.8%)	521 (38.6%)	0.08
5+	2248 (8.5%)	194 (14.2%)	211 (15.6%)	194 (14.4%)	0.04
Off-pump	130 (0.5%)	81 (5.9%)	81 (6.0%)	81 (6.0%)	0.00

*CABG* indicates coronary artery bypass grafting, *SD* standard deviation, *CC* creatinine clearence, *COPD* chronic obstructive pulmonary disease, *PCI* percutaneous coronary intervention, *MI* myocardial infarction; Standardized difference = |P_NT_ − P_C_|/√((P_NT_ (1 − P_NT_) + P_C_ (1 − P_C_))/2)

### Mortality

Thirty-day mortality was 0.3% for the NTT group and 0.9% for the CT group. Mortality occurred in 14.5% (196/1349) of NTT patients and 14.5% (195/1349) of CT patients during follow-up. The hazard ratio for death adjusted for risk factors for NTT as compared to CT was (95% CI) 0.97 (0.80–1.19) (Table 2). The full regression model is available as online Additional file 1.

**Table 2:** Cox regression for risk of death, first clinically-driven angiography and first reintervention for the NTT group as compared to the CT group

	No-touch	*P*-value
Death		
HR unadjusted (CI 95%)	1.03 (0.79–1.18)	0.76
HR adjusted (CI 95%)	0.97 (0.80–1.19)	0.80
Angiography		
HR unadjusted (CI 95%)	0.80 (0.66–0.97)	0.022
HR adjusted (CI 95%)	0.76 (0.63–0.93)	0.007
Reintervention		
HR unadjusted (CI 95%)	0.95 (0.74–1.22)	0.69
HR adjusted (CI 95%)	0.91 (0.71–1.17)	0.47

Hazard ratio (HR) was adjusted for age (40–50, 50–60,60–70 or 70–80 years), sex, number of distal anastomoses (2, 3, 4 or 5+), BMI (< 25, 25–30 or > 30), diabetes status, kidney function (creatinine clearence > 85, 50–85, < 50 or dialysis), COPD, neurologic disability, left ventricle ejection fraction (normal, 30–50, < 30%), MI prior to operation, previous PCI, urgency of operation and use of cardiopulmonary bypass

### Clinically-driven angiography

Post-operative clinically-driven angiography occurred in 14.0% (189/1349) of NTT patients and 16.7% (225/1349) of CT patients during follow-up. The hazard ratio for first angiography adjusted for risk for NTT as compared to CT was (95% CI) 0.76 (0.63–0.93) (Table 2).

### Reintervention

There was a need for reintervention in 8.9% (120/1349) of NTT patients and 8.3% (122/1349) of CT patients during follow-up. The hazard ratio for need for reintervention adjusted for risk factors for NTT as compared to CT was (95% CI) 0.91 (0.71–1.17) (Table 2).

### Indications for angiography and occurrence of failed grafts

STEMI was the indication for the first angiography 7.4% of NTT patients and 5.8% of CT patients (Table 3). Failed grafts were found at first angiography in 43.2% of NTT patients and 53.6% of CT patients (Table 3).

**Table 3:** Total number of patients with clinically-driven angiography during follow-up. Indications for the first angiography and the fraction of patients with failed grafts found during the procedure for the NTT and CT group

	Conventional	No-touch
Total number	225	189
Indications		
STEMI	5.8%	7.4%
NSTEMI or unstable angina	40.9%	47.1%
Stable angina	44.0%	39.7%
Other	9.3%	5.8%
Graft failure		
Failed grafts	53.6%	43.2%
No data	7.1%	3.2%

STEMI indicates ST-segment elevation myocardial infarction; NSTEMI non ST-segment elevation myocardial infarction

## Discussion

In this study NT grafting was associated with a lower risk for repeat angiography, however no difference could be observed for mortality and need for reintervention. Earlier reported improvements in patency of NT veins are possibly reflected in an improved clinical outcome during the first 10 years after surgery.

Although the NT technique has been used in practice for more than 15 years, the evidence for this method is limited to studies with a small number of patients that have demonstrated improved angiographic patency. It has been unknown how this apparent improved patency translates into an improved clinical outcome. Main determinants of vein graft failure has been proposed to be a small vessel diameter, reduced wall motion of the vessel-dependent myocardial region and the right coronary as target vessel [10]. This indicates that it often are grafts supplying less important myocardial regions that fail and perhaps why they generally do so without any symptoms and seemingly without increasing the mortality or risk of myocardial infarction [8]. An improvement in overall patency does therefore not necessarily confer a better clinical result and may explain the lack of improvements in outcome with multiple arterial grafts [21, 22]. In the first randomised angiographic study of the NT vein, the patency in the group with conventional harvesting technique was high and the difference in patency between groups was initially small [12]. This difference later increased gradually but was not significant and clinically relevant until the 8.5 and 16 years follow-up [16, 18]. The mean follow-up in this study may therefore be too short to detect differences in clinical events.

**Fig. 2:**
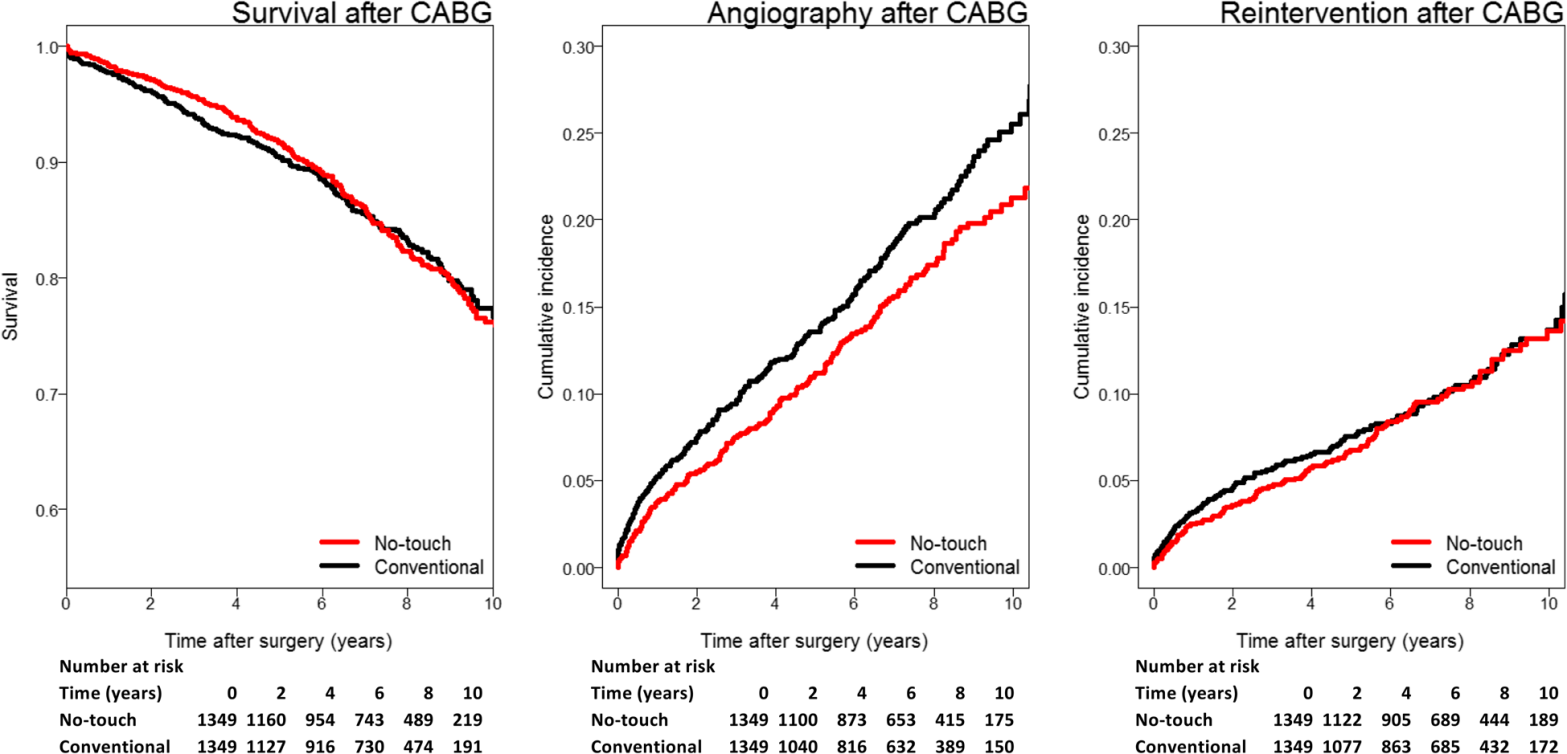
Survival, cumulative incidence of first angiography and first reintervention after CABG for the NT and C group. Tables of number of patients at risk are also shown

There are large regional variations in Sweden how many CABG procedures are performed per number of inhabitants (Fig. 2). Although partly explained by demographics and differences in incidence of coronary artery disease (CAD) in the population, a substantial part of the variation is the result of a difference in the indications and threshold for referral for surgery versus PCI. According to the 2015 annual report from the SWEDEHEART registry, the number of patients referred for CABG surgery in Örebro ranged between 25.4 and 28.2 per 100,000 inhabitants, which is close to the national average of 26.3. The incidence of angiography may reflect the return of CAD symptoms but is highly influenced by other factors such as rates of surveillance with functional studies and the physician threshold for repeat angiography versus optimization of medical therapy. Surgeons in the Örebro region could be performing technically better and medical therapy given to patients after surgery that could influence long-term results [23] is possibly subjected to regional differences. There is thus a possibility for regional variability during follow-up that could introduce confounding. The vast majority of patients in Örebro were operated with the NT technique making the patients receiving conventional vein grafts in this institution a highly selected population depending on several factors not included in the baseline data. Conventional vein harvesting is more often preferred in more fragile patients where quick and simple surgery is the priority, especially in women in whom the harvesting of NT veins might be technically more demanding. NT is favored in patients with expected long survival, but also in patients with coronary vessels of poor quality. In order to avoid confounding patients with conventional vein grafts operated in Örebro were therefore not included in the analysis. Neither the configuration of the grafts nor the targeted vessels or their degree of obstruction can be identified in the registry. There was no formal definition of an occluded or significantly stenosed graft and it was completely operator-dependent. Data on graft failure was not available on an individual graft level and there was no information from the angiography procedure regarding number of failed grafts, their targets or the graft type. It is impossible to establish when a graft failed, what type it was or if the presence of the failed graft in any way was a contributing cause of the symptoms preceding the angiography.

In the present registry-study we use incidence of first clinically-driven angiography and need for repeat intervention as endpoints for evaluation long-term results as they are believed to have a high correlation with symptoms of CAD including symptomatic graft failure. An improvement in the risk for new angiography could be seen for the NT group but not for the need of repeat intervention or survival. The distribution of different indications for the angiography was similar between the groups supporting the view that the incidences of angiography and reintervention in the two groups are similarly linked to an incidence of new coronary events and return of CAD symptoms. Failed grafts were less commonly found during angiography of NT patients as compared to patients with conventional grafts. A smaller proportion of patients in the NT group seem to return with CAD symptoms relating to pathology in the grafts or with concurrent asymptomatic graft failures further supporting the findings in this study and in earlier angiographic studies of NT veins. The study design does, however, not exclude the possibility of residual confounding and a randomized multicentre study needed to confirm these results is being planned.

## Conclusions

In the current study we present follow-up data after CABG from a large Swedish data registry covering all 8 centres. We evaluated results of CABG patients operated in Örebro using the NT technique for vein harvest, comparing outcome with a propensity score matched cohort of patients operated with a conventional harvesting technique in other centres in Sweden. An improvement in the risk for new angiography for the NT group compared to the group with conventional vein harvesting could be observed, however no difference could be observed for mortality and need for reintervention. Earlier reported improvements in patency of NT veins are thereby possibly reflected in an improved clinical outcome during the first 10 years after surgery. A randomized study is needed to confirm these results.

## Additional file

Additional file 1: **Figure S1.** Multivariable adjusted hazard ratios and 95% confidence intervals for death, first angiography, and first need for reintervention. CABG indicates coronary artery bypass grafting; IMA internal mammary artery; SVG saphenous vein graft; CI confidence interval; CC creatinine clearance; COPD chronic obstructive pulmonary disease; PCI percutaneous coronary intervention; MI myocardial infarction. (TIF 4141 kb)

## Abbreviations

BIMA: Bilateral internal mammary artery
BMI: Body mass index
CABG: Coronary artery bypass grafting
CAD: Coronary artery disease
CC: Creatinine clearance
COPD: Chronic obstructive pulmonary disease
IMA: Internal mammary artery
MI: Myocardial infarction
NT: “no-touch”
PCI: Percutaneous coronary intervention
SVG: Saphenous vein graft
