# Supplementary figures and images for: Long-term clinical outcomes after coronary artery bypass grafting with pedicled saphenous vein grafts
Source: J Cardiothorac Surg. 2018 Nov 26;13:122. doi: 10.1186/s13019-018-0800-z (PMC6258143; doi:10.1186/s13019-018-0800-z)

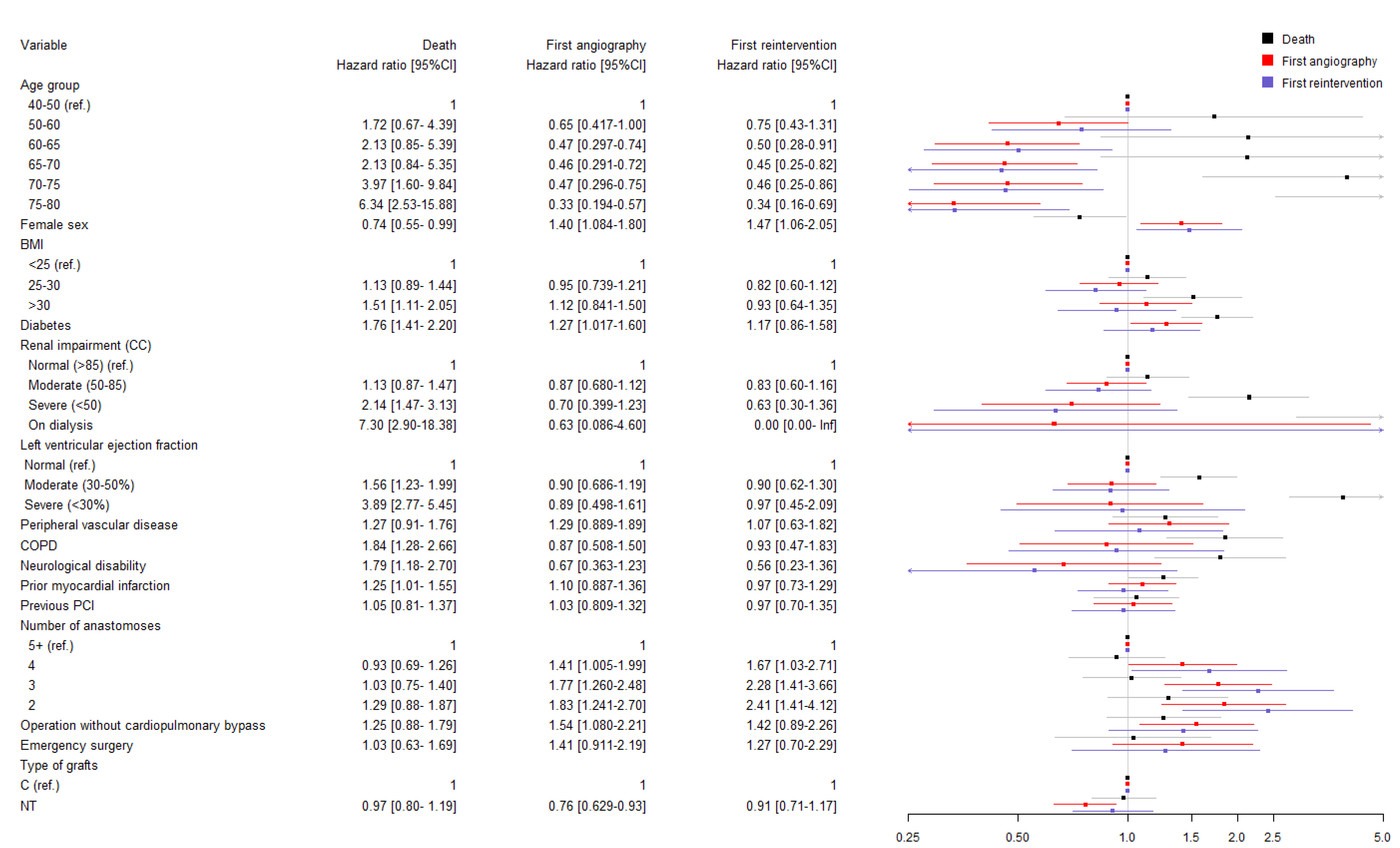

Supplement: Supplementary file 1 — Figure S1. Multivariable adjusted hazard ratios and 95% confidence intervals for death, first angiography, and first need for reintervention. CABG indicates coronary artery bypass grafting; IMA internal mammary artery; SVG saphenous vein graft; CI confidence interval; CC creatinine clearance; COPD chronic obstructive pulmonary disease; PCI percutaneous coronary intervention; MI myocardial infarction. (TIF 4141 kb) [file 13019_2018_800_MOESM1_ESM.tif]
